# Morphologies of Comb-like Polyacrylic Acid/Polyacrylate Copolymers as Functions of the Degree of Derivatization with *n*-C_22_H_45_ Side Chains

**DOI:** 10.3390/polym15244663

**Published:** 2023-12-11

**Authors:** Tomoya Okada, Mizuho Ishii, Harumi Sato, Go Matsuba

**Affiliations:** 1 Graduate School of Organic Materials Engineering, Yamagata University, 4-3-16 Jonan, Yonezawa 992-8510, Yamagata, Japan; 2 Graduate School of Human Development and Environment, Kobe University, Kobe 657-8501, Hyogo, Japan; hsato@tiger.kobe-u.ac.jp

**Keywords:** grafted copolymer, crystal morphology, structural analysis

## Abstract

Polymers with crystallizable side chains have numerous applications, and their properties depend on their crystal morphologies and phase separation. Structural analysis on a wide spatial scale plays an important role in controlling the thermal properties and higher-order structures of these polymers. In this study, we elucidated the melting and crystallization processes of copolymers with varying crystallizable side-chain fractions over a wide spatial range. Differential scanning calorimetry revealed that the enthalpies of melting and crystallization increased linearly with increasing crystallizable side-chain fraction. The results of wide-angle X-ray scattering indicated that the crystal lattice was hexagonal. Conversely, spherulite-like higher-order architectures with linear structures and radial spreading were observed in the highly crystallizable components, but no micrometer-scale structures were observed in the less crystallizable components. In situ small-angle X-ray scattering was used to elucidate the phase separation and mixing processes. Lamellar crystallites were observed at crystallizable side-chain fractions of >55 wt.%, whereas small crystallites were observed at fractions of <45 wt.%. At temperatures above the order-disorder transition temperature, density fluctuations caused by correlation holes were observed. These properties have a strong effect on the crystallizable side-chain fraction.

## 1. Introduction

Comb-like polymers, which comprise polymer main chains and pendant side chains, exhibit characteristic crystalline structures, self-assembly morphologies, and melt properties [1,2,3,4]. Accordingly, their crystallization and segmental packing differ from those of typical linear polymers because of the influences of their structural compositions and side-chain lengths [5,6,7,8,9,10]. Consequently, the manipulation of comb-like polymers, which exhibit different behaviors in terms of side-chain crystallization, is useful in understanding their local chain-packing modes [6,11,12,13,14,15]. In addition, the polymer backbone, side-chain length, and chemical linkages significantly influence the characteristic chain packing and side-chain crystallization of a comb-like polymer. When longer side chains are introduced along the polymer backbone, an ordered packing structure is typically formed because of the increased number of crystallizable CH_2_ units [16,17].

The attachment of appropriate side chains to their polymeric backbones may result in materials with remarkable structures and advanced functionalities, such as charge transport, in addition to optical properties and responsive performance [12,18]. Understanding the aggregated structures of comb-like polymers is advantageous in designing responsive materials with novel architectures. Furthermore, the pendant side chains do not fully participate in crystallization, and only the C atoms that are positioned away from the main chain enter the crystalline phase [4,16]. Thus, the minimum requirement or critical number of crystallizable C atoms varies with the stiffness of the polymer backbone [19,20]. In addition, the lengths of the side alkyl groups determine the crystal domains of the comb-like polymers, which exhibit distinct structural and packing patterns. Previous research regarding *n*-alkylated poly(*p*-benzamide) [21], polyethyleneimine [22], chitosan [23], and polyvinyl alcohol [24] comb-like polymers with side-chain lengths ranging from 12 to 22 carbon atoms showed that the melting temperatures (*T*_m_) and enthalpies (Δ*H*_m_) of the paraffin-like crystallites depend strongly on the sizes of the alkyl domains.

The comb-like polymer used in this study exhibits the same chemical structure as Intellimer^TM^, which is an acrylic adhesive [25,26]. Intellimer^TM^ is under development for use as a thermosensitive adhesive based on the significant changes in its various properties with temperature. It is also under consideration for use as a film whose transparency and opacity may be controlled with temperature. Its physical properties have been summarized in several studies [26,27]. In this study, we used a semi-crystalline graft copolymer of short- and long-chain esters of polyacrylic acid, with polyacrylic acid as the main-chain backbone. The long-chain ester, which behaves as a side chain, contains the linear alkyl group C_n_H_(2n + 1)_, which crystallizes at low temperatures. Heating to the melting point of the linear alkyl moiety causes a transition from a semi-crystalline to an amorphous state. This transition may modulate properties such as the thermosensitive viscosity, permeability, and adhesion.

Therefore, by manipulating the primary structures of the crystallizable side chains, it is possible to vary the temperature at which the phase transition between crystallization and melting occurs, i.e., to change the adhesion and release properties. Additionally, the melting point and viscoelastic properties may be altered by varying the side-chain density. Such adhesives find numerous applications in the field of electronics, including in lithography [28] and the fabrication of indium gallium zinc oxide panels [29,30,31]. In addition, as their viscoelasticities vary with temperature, they are used in microcapsule coatings for seeds, with an emphasis on sustainability [32,33], and in films to control permeability to air [34]. Additionally, graft polymerization of materials such as Intellimer^TM^ onto the surfaces of microparticles to generate temperature-dependent microparticles has been studied [35]. The relationship between the crystal structure of a comb polymer and its adhesive properties has been elucidated via detailed analysis of the crystal structure, of changes in the higher-order crystal structure with temperature, and of the adhesion and release properties of the material [36]. In this study, thermal measurements and spatially extended structural analyses were used to evaluate the crystallization and melting processes of comb polymers with different weight percents of crystallizable side-chain components. The fraction dependences of the crystalline components are the focus of this study. In situ wide-angle X-ray scattering (WAXS) and synchrotron radiation were used to evaluate crystal growth and melting, and we evaluated their sub-micrometer structures using in situ small-angle X-ray scattering (SAXS). Depolarized light scattering and polarized light and atomic force microscopy (AFM) were used to study the micrometer-scale structure of the material. Accordingly, the degree of crystallinity, crystal size, density fluctuation, and higher-order structure as functions of temperature were elucidated and summarized.

## 2. Materials and Methods

### 2.1. Materials

**Figure 1:**
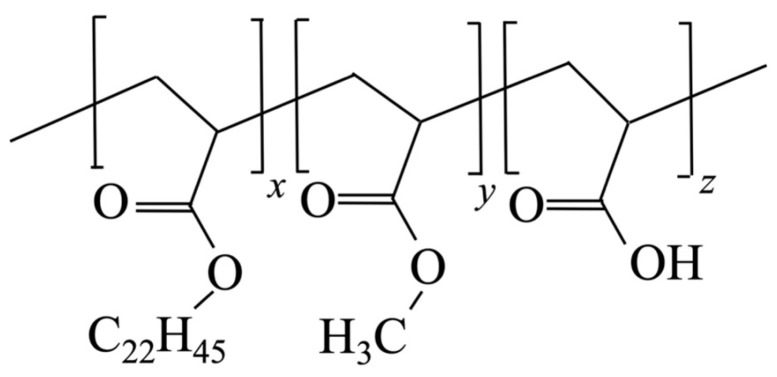
Simplified chemical structure of the random polyacrylic acid-type copolymer with *n*-C_22_H_45_ crystallizable side-chain components.

Figure 1 shows a simplified chemical structure of the graft copolymer consisting of acrylic acid, methyl acrylate, and monomers randomly grafted with acrylic acid-linked alkyl side chains. The main chains, which consist of polyacrylic acid, poly(methyl acrylate), and 1-docosanol (C_22_H_45_OH)-linked polyacrylic acid, were supplied by Nitta, Osaka, Japan. *n*-Alkyl C_22_H_45_, as shown in Figure 1, crystallizes. The graft copolymer Intellimer^TM^ is a trademark of Landec (Menlo Park, CA, USA), which has a commercial licensing partnership with Nitta. The ratio of acrylic acid monomers to side-chain C_22_H_45_/CH_3_/H was controlled by mass. Table 1 shows the proportions of 1-docosanol acrylate, methyl acrylate, and acrylic acid employed. The weight-averaged molecular weight for all samples is about 500,000.

### 2.2. Measurements

Differential scanning calorimetry (DSC) was performed using a DSC Q200 (TA Instruments, New Castle, DE, USA), which was calibrated using the *T*_m_ values of In and cyclohexane standards. Samples of ~5 mg were heated in Al pans from 0 to 100 °C at a rate of 10 °C/min. The melting or crystallization temperature (*T*_m_ or *T*_x_) is the onset temperature of endothermy or exothermy.

SAXS and WAXS were performed using the BL-6A beamline (Photon Factory, High Energy Accelerator Research Organization, Tsukuba, Japan) at a wavelength of 0.15 nm [37,38]. The respective camera lengths used in SAXS and WAXS were 931 and 243 mm, and the respective detectors used in SAXS and WAXS were PILATUS 1M and PILATUS 100K (DECTRIS, Baden, Switzerland). The ranges of the scattering vector *q* (=4πsinθ/λ, 2θ: scattering angle, λ: wavelengths used in SAXS and WAXS) were from 6.0 × 10^−2^ to 2.5 and 8 to 19 nm^−1^, and the thickness of each sample was approximately 200 μm. During in situ SAXS and WAXS, the sample film was sandwiched between two polyether ether ketone films and mounted on a Linkam 10002L hot stage (Linkam Scientific Instruments, Redhill, UK) for temperature control. FIT2D software (version 12.077, European Synchrotron Radiation Facility, Grenoble, France) was used for data processing, including contrast control of the 2D patterns and the creation of 1D profiles of the obtained 2D patterns.

AFM was performed to observe the internal structure on the sub-micrometer scale using an Agilent 5500 (Agilent Technologies, Santa Clara, CA, USA). The tapping mode was used to measure the topography, and the phase contrast was measured. The probes used were PPP-NCL probes (NANOSENSORS, Neuchatel, Switzerland) with respective spring constants and resonance frequencies of ~50 N/m and ~190 kHz, yielding a respective scan size and image resolution of 20 × 20 nm and 256 × 256. After the samples were bonded to Si substrates, Kapton films were applied to their surfaces.

Depolarized small-angle light scattering using a PP-1000 system (Otsuka Electronics, Osaka, Japan) was performed to observe the micrometer-scale higher-order structures. The camera length was 50 mm, and the light source was a He-Ne laser (λ = 654 nm). The range of the scattering vector *q* was 0.30–2.0 μm^−1^, and the sample, with a thickness of ~40 μm, was sandwiched between two cover glasses. The temperature was controlled using the Linkam 10002 L hot stage [39]. Optical microscopy was performed using a VW-5000 microscope (Keyence, Osaka, Japan) equipped with high-speed charge-coupled device (CCD) camera attachments [40].

Raman spectra were measured in the 400–100 cm^−1^ range using a LabRAM HR Evolution (HORIBA, Kyoto, Japan) equipped with a Syncerity CCD detector at an excitation wavelength of 532 nm. The respective spectral resolution, laser power, exposure time, and number of scans used in Raman spectroscopy were 0.5 cm^−1^, 100 mW, 15 s, and 5. Temperature-dependent Raman spectroscopy was performed using a T95-HS temperature controller (Linkam Scientific Instruments) [41].

## 3. Results and Discussion

### 3.1. Thermodynamic Phenomena

**Figure 2:**
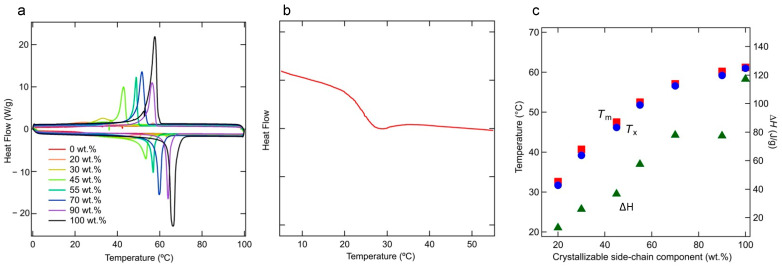
Effects of fraction on the thermal properties. The DSC thermograms reveal crystallization during cooling from the molten state and melting due to heating of the crystalline/amorphous material between 0 and 100 °C (heating and cooling rates = 10 °C/min (**a**). Magnified DSC thermogram of the 0 wt.% sample (**b**). Melting (*T*_m_; solid squares) and crystallization temperatures (*T*_x_; solid circles) and melting enthalpies (solid triangles) at different fractions (**c**).

Figure 2a shows the DSC thermograms obtained at cooling and heating rates of 10 °C/min. In the thermogram of the 0 wt.% sample, *T*_m_ and the crystallization temperature *T*_x_ are not observed; thus, no crystallization occurs in this sample. In contrast, *T*_x_ and *T*_m_ increase as the fraction of crystallizable side-chain components increases above 20 wt.%. Figure 2b shows that the glass transition temperature of the 0 wt.% sample is approximately 24.2 °C. Figure S1 and Table S1 shows DSC thermograms of 55 wt.% for the definition of *T*_m_ and *T*_x_ and the peak temperature and finishing temperature for melting and crystallization. The correlations between the content of the crystallizable side-chain component and *T*_m_ and *T*_x_ are shown in Figure 2c. Even at 45 wt.%, the *T*_m_ exceeds that of 1-docosane (C_22_H_46_) crystals (43.9 °C) [42]. The higher melting point may be due to the attachment of the crystallizable side chains to the main chain, which hinders molecular motion. The enthalpy (Δ*H*) and the crystallization enthalpy increase as the weight percent of the crystallizable side-chain component increases [43].

### 3.2. High-Ordered Structures at the Submicron and Micron Scales

**Figure 3:**
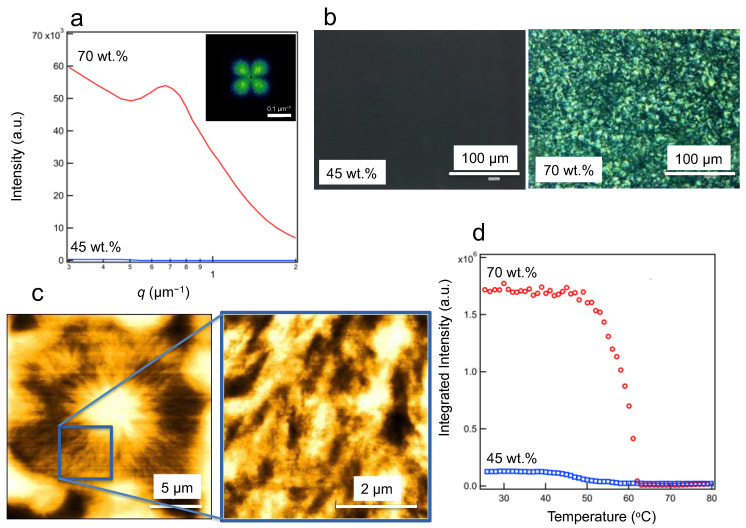
Micrometer-scale structures of the samples at different fractions. (**a**) Depolarized light-scattering profiles of the 70 and 45 wt.% samples and 2D scattering profiles of 70 wt.% sample. (**b**) Polarized optical micrographs of the 70 and 45 wt.% samples. (**c**) AFM images of the 70 wt.% sample at different magnifications, showing the spherulite-like structure and a magnified view of the linear structure. (**d**) Temperature dependence of the integrated scattering intensity of the depolarized light.

Figure 3a,b shows the depolarized small-angle light-scattering profiles and polarized optical micrographs of the 45 and 70 wt.% samples at 25 °C. Below 45 wt.%, a very low scattering intensity was observed, indicating that the microscale structure forms slowly, whereas at 70 wt.%, a peak was observed at about *q_p_* = 0.7 μm^−1^. Figure 3c shows 10 μm-scaled spherulite-like structures under 70 wt. % conditions, as observed via AFM with linear structures. From the depolarized small-angle scattering pattern, an average spherulite size, *R*_s_, was calculated from the peak position, *q*_p_, using Equation (1) [44,45].

(1) $$R_{s}=\frac{4.09}{q_{p}},$$

Here, *q*_p_ is the magnitude of the scattering vector at the peak position in the four-leaf clover patterns in the inset of Figure 3a. Therefore, the *R*_s_ was 5.8 μm. Figure 3d shows the temperature dependence of the total integrated scattering intensity during heating. Scattering is no longer observed upon melting. The temperature at which scattering completely disappears coincides with the melting point measured using DSC, indicating that these higher-order architectures display crystalline-derived oriented structures.

### 3.3. Crystal Lattice Determined via WAXS

**Figure 4:**
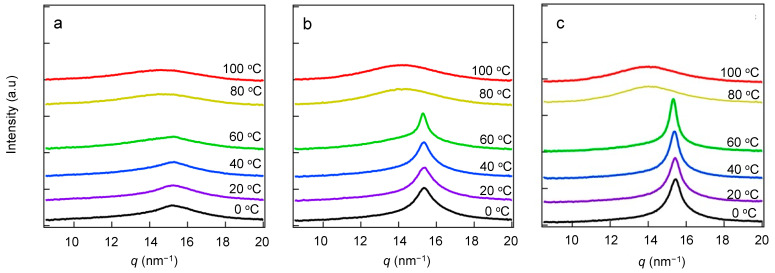
Temperature dependences of the in situ WAXS profiles of the 30 (**a**), 55 (**b**), and 70 wt.% (**c**) samples at a heating rate of 10 °C/min during heating of their crystalline/amorphous states from 0 °C. The baseline of each profile is shifted up or down to avoid overlap.

Figure 4a–c shows the results of WAXS of copolymers with different side-chain fractions following heating of their crystalline states. In the profiles of all samples, we observed that the diffraction peak at *q* = 15.3 nm^−1^ starts to decrease above *T*_m_ because the crystals are melting. Therefore, these peaks were considered with respect to the crystal structure of the crystallizable side-chain component.

Figure S2 shows the results of WAXS of the copolymers at 0 °C. We could find no crystalline peak for the crystallizable side-chain component of 0 wt.%, with only an amorphous halo observed; thus, the main chain may not crystallize above 0 °C. If the alkyl portion of the side chain exhibits the same orthorhombic structure as the polyethylene crystal, crystal-diffraction peaks should be present, in addition to the amorphous halo. However, only one amorphous halo and one diffraction peak were observed in this study. For a detailed analysis, we calculated the correlation length *d* based on the peak position using Equation (2).

(2) $$d=\frac{2\pi }{q_{peak}},$$

where *q*_peak_ is the peak position in the scattering profile. Spacing values *d*_WAXS_ of 0.42 nm were observed in the hexagonal structures of the alkyl chains [11,46,47]. During heating, the diffraction peak representing the hexagonal crystalline structure decreases and shifts based on the smaller angle, and only an amorphous halo at *q* ≈ 14.0 nm^−1^ is observed when the material is melted. We compared the crystallinities at different temperatures. The ratio of the crystalline regions in the WAXS profiles, *R*_c_(*T*), is defined in Equation (3).

(3) $$R_{c}\left(T\right)=\frac{I_{cry}\left(T\right)}{I_{cry}\left(T\right)+I_{amo}(T)},$$

where *I*_cry_(*T*) and *I*_amo_(*T*) are the respective areas of the crystalline and amorphous portions of the WAXS profiles. Figure S3 shows the decomposition of the 70 wt.% sample at 0 °C based on the WAXS profile. *R*_c_(*T*) depends on the crystallinity, and we evaluated the crystalline size, *L_hkl_*, using the Scherrer equation (Equation (4)).

(4) $$L_{hkl}=\frac{k\lambda }{\beta \cos\theta }$$

**Figure 5:**
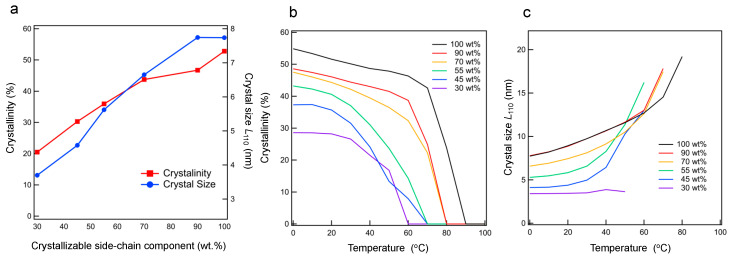
(**a**) Ratio *R*_c_(*T*) of Equation (2) and crystal size *L*_110_ of the Scherrer equation (Equation (3)), as determined based on the in situ WAXS profiles of the samples with different side-chain fractions at 0 °C. (**b**) *R*_c_(*T*) and (**c**) *L*_110_ at different temperatures during heating of the crystalline/amorphous states of the samples from 0 °C.

Figure 5a shows the *R*_c_(*T*) and *L*_110_ of the side-chain crystalline component at 0 °C, which is below the melting point. *R*_c_(*T*) increases with increasing fraction. In particular, in the case of 70 and 90 wt.%, *R*_c_(*T*) increases slightly. These results are consistent with the results for Δ*H* from DSC thermograms in Figure 2c. Similarly, *L*_110_ increases with increasing fraction.

Figure 5b,c shows the respective temperature dependences of *R*_c_(*T*) and *L*_110_ during heating. *R*_c_(*T*) decreases with increasing temperature, becoming zero above the melting point determined via DSC. At fractions above 45 wt.%, *L_hkl_* increases with increasing temperature, while *R*_c_(*T*) decreases with temperature. These results suggest that the increase in *L*_110_ is due to the aggregation and enlargement of small crystals with partially melting side-chain crystals. However, at a low fraction (30 wt. %), *L*_110_ is independent of temperature, even at temperatures close to the *T*_m_. Linear long-chain organic molecules are known to form lamellar intermediate phases (also called rotator phases) between their fully ordered crystalline phases and their isotropic liquid phases. The review by Cholakova and Denkov suggested that endothermic or exothermic peaks were observed during phase transition of the rotator phase of *n*-alkane [48]. Even in graft copolymers, endothermic peaks were observed [49]. We discuss the rotator phase between *T*_m_ and *T*_m_finish_. From the report by Cholakova and Denkov [50], the difference between the melting point and the transition temperature of the rotator phase of C_22_H_46_ is 1.4 °C. On the other hand, the difference between *T*_m_ and *T*_m_finish_ is between 10.4 and 16.3 °C in Table S1. These data suggest that both the transition from fully crystalline to rotator phase and the transition from the rotator phase to isotropic melt occur over a wide temperature range in the case of comb-like copolymers, making it difficult to distinguish between these two translon processes. Consequently, one endothermic peak was observed in Figure 2a.

### 3.4. Sub-Micrometer-Scale Structure Determined via In Situ SAXS

**Figure 6:**
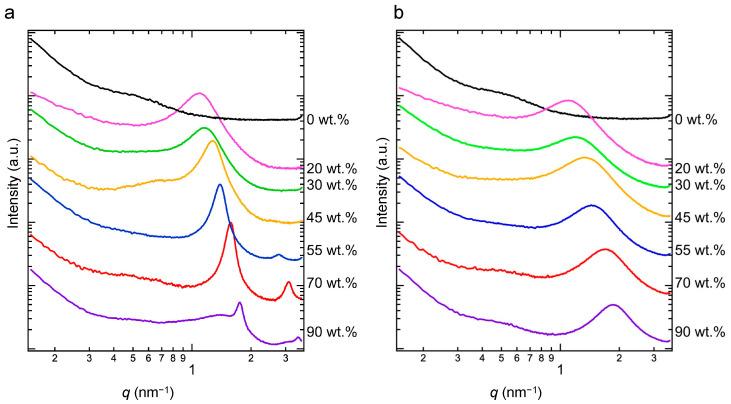
In situ SAXS profiles obtained at 0 (**a**) and 100 °C (**b**). The baseline of each profile is shifted up or down to avoid overlap.

Figure 6a,b shows the SAXS profiles obtained at 0 and 100 °C, respectively. As shown in Figure 6a, the peak at *q* ≈ 1.5 nm^−1^ shifts to a higher *q* as the fraction of the crystalline alkyl side chain increases, and the profile of the 0 wt.% sample displays very weak peaks. Therefore, the peak represents the ratio of the polyacrylic acid main chains to crystalline alkyl side chains.

Meanwhile, as shown in Figure 6b, broad peaks were observed at *q* ≈ 1.5 nm^−1^ and the secondary peaks are no longer observed. The correlation length *d* is calculated based on the peak position *q*_peak_ of the SAXS profile using Equation (1), and Table 2 shows the correlation lengths *d* at 0 and 100 °C. Above 30 wt.%, the correlation length at 100 °C is smaller than that at 0 °C. The correlation length decreases with an increase in the content of the crystallizable side-chain component. Based on the DSC thermograms, the crystallinity increases as the content of the crystallizable side-chain component increases. The correlations calculated from SAXS measurements are those between crystals and crystallites or between crystalline and amorphous components. A low crystallizable side-chain component weight percentis associated with a low a correlation length between crystals. In other words, the increase in the correlation length with a decreasing crystalline component weight percent is due to the increase in the amorphous component, which increases the thickness of the amorphous region between crystals and thus the distance between crystals.

**Figure 7:**
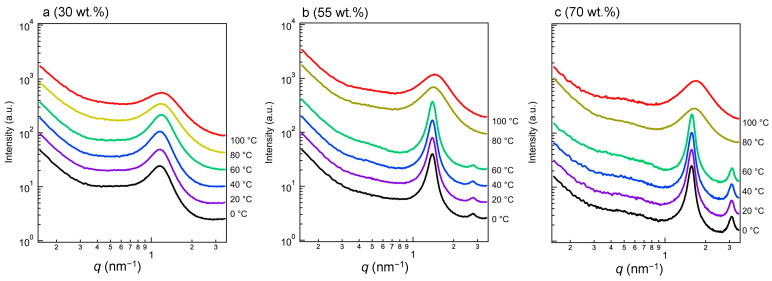
Temperature dependences of the in situ SAXS profiles of the samples with contents of the crystallizable side-chain component of 30 (**a**), 55 (**b**), and 70 wt.% (**c**) at a heating rate of 10 °C/min during heating from their crystalline/amorphous states from 0 °C. The baseline of each profile is shifted up or down to avoid overlap.

Figure 7a–c shows the temperature dependences of the SAXS profiles of the samples with crystalline alkane side-chain fractions of 30 (a), 55 (b), and 70 wt.% (c) during heating from 0 to 100 °C. There is a sharp peak at *q* = 1.5 nm^−1^ due to the crystalline phase. As shown in Figure 7b,c, a secondary peak is observed close to *q* = 3.0 nm^−1^ at temperatures below that at which the crystals are completely melted, *T*_m_finish_ in Table S1. Figure 6a shows the same secondary peaks in the profiles of the 45, 55, 70, and 90 wt.% samples. These results suggest that these copolymers contain lamellar microdomains with sizes of 3.59–4.62 nm. The correlation length is 1.3~1.6-fold longer than the fully extended crystallizable side-chain length of 2.8 nm. Thus, the SAXS profiles indicate that the polymer’s main chains form a lamellar plane with the *n*-docosane side chains. We evaluated the crystal thickness of the 100 wt.% sample using SAXS and DSC, and Figure S4 shows the Raman spectra obtained during heating. The longitudinal acoustic vibrations of the molecular alkyl chains were observed using low-frequency Raman spectroscopy.

Above *T*_m_finish_ or at a low crystalline alkane side-chain weight percent, only the broader peak is observed, with no secondary peak, i.e., when the secondary peak is absent, as shown in Figure 7a, the observation of one SAXS peak may be insufficient to support the conclusion that a lamellar morphology has formed within the polymer complex [51,52,53,54,55,56,57,58]. Therefore, we observe small crystallites, even below *T*_m_. At the *T*_m_ of the onset temperature of melting, the peaks of SAXS profiles of the samples becomes weak. At the temperatures at which the melting process finishes (*T*_m_finish_; in Table S1), only single broad peaks are seen due to the disordered states of the polymers. Thus, the complexes may exhibit characteristic copolymer-like fraction fluctuations (correlation hole effects) [53,54,55]. For a more detailed analysis, we focused on the order-disorder transition (ODT) and the melting process at about *T*_m_. The change in the scattering pattern across the ODT enables the determination of the ODT temperature (*T*_ODT_). There are several methods based on mean-field theory of identifying *T*_ODT_ using the results of SAXS. Two of the most common methods used are plots of (i) the reciprocal of the maximum scattering intensity (*I*_m_^−1^ (*q*_m_)) against the reciprocal of the absolute temperature (*T*^−1^) and (ii) the correlation length *d_SAXS_* (=2π/*q*_m_) against *T*^−1^. According to Leibler’s mean-field theory, *I*_m_^−1^ (*q*_m_) should decrease linearly with *T*^−1^ in the disordered state:

(5) $$I_{m}^{-1}\left(q_{m}\right)\sim a-\frac{b}{T},$$

where *a* and *b* are positive constants [56,57,58]. Equation (5) assumes the temperature dependence of the Flory-Huggins segmental interaction parameter between the polyacrylic acid main chain and crystallizable alkane side chains. Leibler’s theory predicts that *d_SAXS_* or *q*_m_ in the disordered state is essentially independent of the temperature if we omit the small temperature variation due to the temperature dependences of the radii of gyration of the copolymer chains *R*_g_(*T*).

(6) $$d_{SAXS}/R_{g}(T)\sim T^{0}.$$

**Figure 8:**
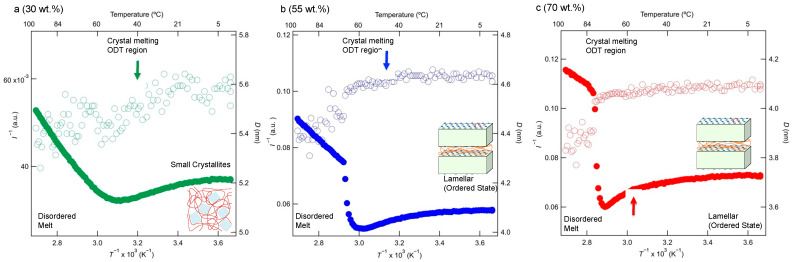
Dependences on the reciprocal of the absolute temperature of the reciprocals of the peak intensities *I*_m_^−1^ (*q*_m_) (filled circles) and correlation lengths *d* (open circles) of the samples with weight percents of the crystallizable side-chain component of 30 (**a**), 55 (**b**), and 70 wt.% (**c**) at a heating rate of 10 °C/min during heating of their crystalline/amorphous states from 0 °C. The arrows indicate the melting points based on the DSC thermograms.

Thus, in the context of mean-field theory, the deviations in the temperature dependences of *I*_m_^−1^ (*q*_m_) in Equation (5) and *d_SAXS_* in Equation (6) are attributed to the onset of the disordering process upon heating, and they may thus be used to determine *T*_ODT_. Figure 8a–c show plots of *I*_m_^−1^ (*q*_m_) and *d* for the different crystallized copolymer components as functions of *T*^−1^. As shown in Figure 8a–c, *I*_m_^−1^ (*q*_m_) and *d_SAXS_* are constant with respect to *T*^−1^ at temperatures < *T*_m_ because the crystals in small crystallites (Figure 8a) and the lamellar structure (Figure 8b,c) prevent an increase in density fluctuations during heating. The gray region in Figure 8 shows the temperature during melting process from DSC measurements in Figure 2a. A linear decrease in *I*_m_^−1^ (*q*_m_) is observed at temperatures above the crystal melting temperature, which is a result consistent with the theoretical predictions of Equation (5). Based on Equation (6), *d* is independent of *T* (or *T*^−1^) but increases slightly with *T*^−1^, and Sakurai and Nandan also observed a slight increase in *d* [51,54]. This increase occurs because the copolymer may exhibit an upper critical solution temperature-type phase separation, and the components of the main chain and crystallizable side chains should be mixed at the molecular level at temperatures > *T*_m_. At temperatures just above *T*_m_, *I*_m_^−1^ (*q*_m_) exhibits a peak, and *d* decreases abruptly when the temperature increases, as shown in Figure 8b,c. This behavior is similar to the temperature dependences of star polymers [59]. The respective *T*_ODT_ values of the 55 and 70 wt.% samples are approximately 69 and 80 °C. Conversely, based on Figure 8a, *T*_ODT_ is 56 °C, which exceeds the *T*_m_ values of the crystallites in the 30 wt.% sample.

### 3.5. Modeling the Large-Scale Structures of the Grafted Copolymers with Crystallizable Side-Chain Components

**Figure 9:**
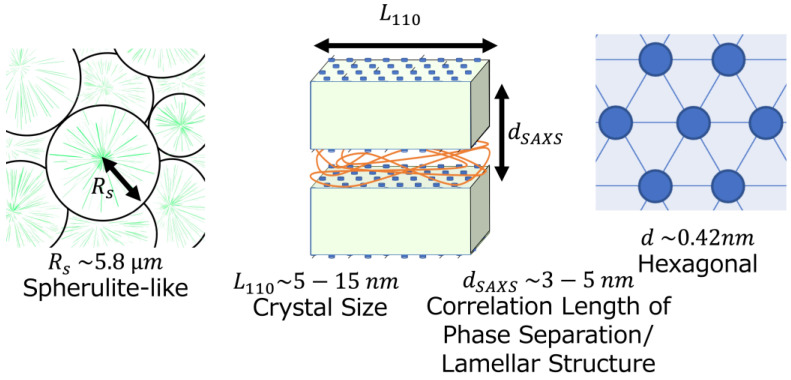
Schematic of the hierarchical structure of the copolymer with side chains with highly crystallizable components and a high weight percent of the crystallizable side-chain component (such as 70 wt.%). The arrows are the sizes of spherulites, *R*_s_, crystal *L*_110_, and correlation of phase separation and lamellar structure, *d*_SAXS_.

Based on these results, models of the crystallizable side-chain copolymers were developed. First, when the weight percent of the crystallizable side-chain component is low (below 30 wt.%), very few crystals were observed, and the morphology depends on the main chain, which is almost randomly coiled. Therefore, small crystallites with low crystallinities were observed. As the crystallizable side-chain weight percent increases, the crystallinities and widths of the crystals also increase. WAXS revealed crystals with hexagonal structures, with correlation values of 0.42 nm. SAXS revealed lamellar structures with sizes of several nanometers in the more crystallizable components, as the correlation length of the lamellar structure is sufficiently smaller than that of the C22 extended chain. Conversely, small crystallites were observed within the less crystallizable components. Above *T*_m_finish_, the crystallites are completely molten and an ODT occurs. Figure 9 shows a schematic summary of the structure of the copolymer. However, when crystallizable side-chain components were present below 20 wt.%, we could not analyze the small-crystallites and phase separation because of very small crystallinity. When the weight percent of the crystallizable side-chain components is above 90 wt.%, the sample amount is very small, making it difficult to analyze the phase separation and crystal morphology for in-situ SAXS/WAXS measurements.

## 4. Conclusions

In situ observations were used to elucidate the melting and crystallization processes of alkyl side-chain polymers with crystallizable side-chain fractions over a broad range of spatial scales. As the weight percent of the crystallizable side-chain component increased, *T*_m_ and *T*_x_ increased. The Δ*H* values of the crystals were also evaluated. In addition, the results of WAXS revealed that the crystal lattice was hexagonal, and SAXS revealed a several-nm-scale lamellar structure with highly crystalline components, as the correlation length of the lamellar structure was sufficiently smaller than that of the C22 extended chain, based partially on Raman spectroscopy below *T*_m_. Between *T*_m_ and *T*_m_finish_, it is difficult to distinguish the processes of transition from crystalline to the rotator phase and melting by DSC. Above *T*_m_, the crystallites were molten. Further, above *T*_m_finish_, an ODT occurred. At a larger scale, a spherulite-like higher-order architecture with a linear structure and radial spreading was observed.

## Figures and Tables

**Table 1:** Ratios (wt.%) of the monomers with side chains of C_22_H_45_/CH_3_/H and ratio of the crystallizable side chain (mol%).

Sample	Monomer (wt.%) with Side-Chain C_22_H_45_/CH_3_/H	Crystallizable Side Chain (mol%)
0 wt.%	0/95/5	0
20 wt.%	20/75/5	5.3
30 wt.%	30/65/5	8.7
45 wt.%	45/50/5	15
55 wt.%	55/40/5	21
70 wt.%	70/25/5	34
90 wt.%	90/5/5	65
100 wt.%	100/0/0	100

**Table 2:** Correlation lengths of the samples with different weight percents of the crystallizable side-chain component annealed at 0 and 100 °C.

Sample	Correlation Length at 0 °C [nm]	Correlation Length at 100 °C [nm]
20 wt.%	5.92 ± 0.05	6.16 ± 0.07
30 wt.%	5.58 ± 0.07	5.44 ± 0.08
45 wt.%	5.08 ± 0.01	4.83 ± 0.08
55 wt.%	4.62 ± 0.02	4.34 ± 0.07
70 wt.%	4.09 ± 0.02	3.85 ± 0.07
90 wt.%	3.59 ± 0.02	3.33 ± 0.07

## Data Availability

Raw data were generated at Yamagata University. Derived data supporting the findings of this study are available from the corresponding author, G.M., on request.

## Supplementary Materials

The following supporting information can be downloaded at: https://www.mdpi.com/article/10.3390/polym15244663/s1, Figure S1: *T*_m_ and *T*_x_ definition from DSC thermograms of 55 wt.% sample; Figure S2: WAXS profiles of different crystallizable side-chain components at 0 °C; Figure S3: The peak decomposition of WAXS profiles of 70 wt.% at 0 °C; Figure S4: Analysis of the temperature dependence of the Raman spectrum of the 100 wt.% sample; Table S1: List of *T*_m_ and *T*_x_, the peak temperature and the finishing temperature during melting and crystallization processes. References [60,61] are cited in the supplementary materials.
