# Supplementary material for: Morphologies of Comb-like Polyacrylic Acid/Polyacrylate Copolymers as Functions of the Degree of Derivatization with n-C22H45 Side Chains
Source: Polymers (Basel). 2023 Dec 11;15(24):4663. doi: 10.3390/polym15244663 (PMC10748179; doi:10.3390/polym15244663)
Supplement: Supplementary file 1 [file polymers-15-04663-s001.zip › polymers-2696907-supplementary.pdf]

Supporting Information

Morphologies of Comb-like Polyacrylic Acid/Polyacrylate Copolymers as Functions of the Degree of Derivatization with *n*-$C_{22}H_{45}$ Side Chains

Tomoya Okada [1], Mizuho Ishii [1], Harumi Sato [2] and Go Matsuba [1,*]

[1] Graduate School of Organic Materials Engineering, Yamagata University, 4-3-16 Jonan, Yonezawa 992-8510, Yamagata, Japan
[2] Graduate School of Human Development and Environment, Kobe University, Kobe 657-8501, Hyogo, Japan; hsato@tiger.kobe-u.ac.jp
* Correspondence: gmatsuba@yz.yamagata-u.ac.jp

Definition of Tm and Tc

Figure S1 shows the DSC thermograms of 55 wt.% sample of the heating rate of 10 °C/min. In this manuscript, we defined T_m and T_x as the onset temperature of melting and crystallization. We evaluated the peak temperature and the finishing ones in Figure S1. Table S1 shows various T_m and T_x.

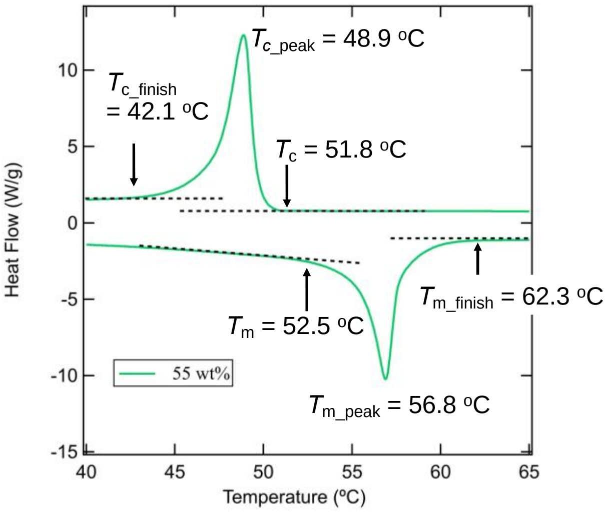

Figure S1. T_m and T_x definition from DSC thermograms of 55 wt% sample.

Table S1. List of T_m and T_x, the peak temperature and the finishing temperature during melting and crystallization processes.

Sample [°C]	T_m	T_{m_peak}	T_{m_finish}	T_x	T_{x_peak}	T_{x_finish}
**20 wt%**	32.6	44.2	48.9	31.7	24.6	17.1
**30 wt%**	40.7	47.5	52.3	39.2	33.2	25.7
**45 wt%**	47.5	53.5	57.9	46.2	42.8	35.6
**55 wt%**	53.3	56.8	62.3	51.8	48.9	42.1
**70 wt%**	57.1	59.7	68.0	56.6	51.5	42.8
**90 wt%**	60.2	63.9	71.8	59.2	56.4	44.5
**100 wt%**	61.2	66.5	75.4	61.0	57.7	45.2

WAXS profiles below T_m

We focused on crystallinity at 0 °C for the different crystallizable side-chain component. In Figure S2, the crystal diffraction peak was observed at 15.3 nm^{-1} in the case of >20 wt.%, while we could observe no crystal diffraction peak of 0 wt.% because of no crystallizable components.

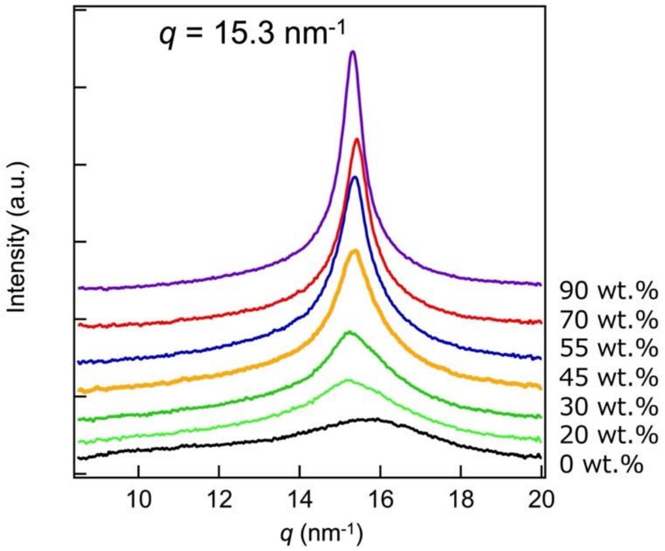

Figure S2. WAXS profiles of different crystallizable side-chain component at 0 °C.

Peak decomposition of WAXS profiles

Figure S3 shows the peak decomposition of WAXS profile of 70 wt.% at 0 °C. The peak at 15.4 nm^{-1} is assigned as the diffraction from crystalline, and the broad peak is due to amorphous halo.

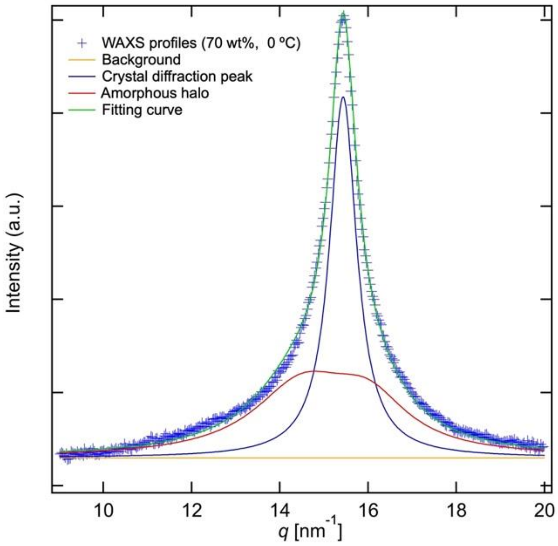

Figure S3. WAXS profiles of 70 wt.% at 0 °C (blue cross). The background, crystal diffraction peak, and amorphous halo are orange, purple, and red lines, respectively. The fitting curve is green line.

Low frequency Raman spectra of 100 wt.% sample

We focused on 100 wt.% crystallizable component sample of crystal structure. The enthalpy of crystal melting of the 100 wt% crystallizable component sample was ~117.2 J/g (=36.4 kJ/mol) in Figure 2a. Because the enthalpy of crystal melting of 1-docosane was 158.4 J/g (=49.0 kJ/mol) based on the published data [S1], the crystallinity was estimated to be 74% These findings imply that the entire side-chain component could not be crystallized even in 100 wt% sample. From SAXS measurements, the crystal thickness is evaluated to be ~1.96 nm in a 100 wt% case. The correlation length crystal alkane chain would be composed of $C_{15.4}$.

Furthermore, Figure S4 shows the Raman spectra of 100 wt% sample during heating up. The peak of the Raman shift was observed at 160 cm^{-1}. This result suggests that the longitudinal acoustic mode (LAM), "accordion", vibration of *n*-alkyl side chains. The Raman shift is consistent between C15 and C16 [S2]. The peak disappears at 80°C above T_m.

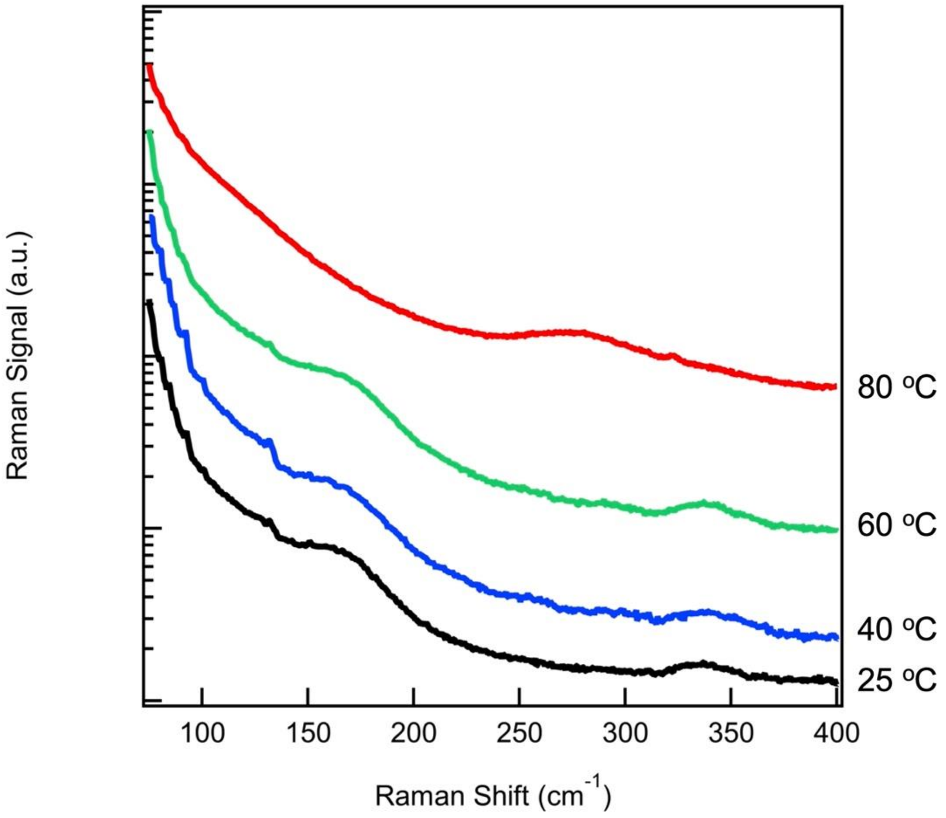

Figure S4. Raman spectra of 100 wt.% crystallizable side-chain sample during heating up.

References:

[S1] P. Claudy, J.M. Letoffe, Phase transitions in even n-alkanes CnH2n+2, n = 16-28. Characterization by differential calorimetric analysis and by thermooptical analysis. Effect of deuteration. *Calorim. Anal. Therm.,* **1991,** *22,* 281.

[S2] R. F. Schaufele, Chain Shortening in Polymethylene Liquids, *J. Chem. Phys.*, **1968**, *49*, 4168.
